# Managing Pregnancy and Nursing Affecting African American Women with Inflammatory Bowel Disease: Clinical Outcomes and Parenthood

**DOI:** 10.18103/mra.v11i6.3784

**Published:** 2023-06-26

**Authors:** Amosy E. M’Koma, Jamie N. Ware, Rosemary K. Nabaweesi, Sanika S. Chirwa

**Affiliations:** 1 Departments of Biochemistry, Cancer Biology, Neuroscience and Pharmacology; 2 Departments of Obstetrics and Gynecology; 3 Center for Health Policy, Meharry Medical College, Nashville, TN 37208, USA.

**Keywords:** Pregnancy, inflammatory bowel disease, Healthcare system inclusiveness, insurance policies, systemic-institutionalized-structural-racism

## Abstract

Inflammatory bowel disease (IBD) is a term for two autoimmune diseases encompassing Crohn’s disease (CD) and ulcerative colitis (UC) which are lifelong diseases affecting more than 3 million adults (1.3%) in the United States. IBD is characterized by chronic inflammation of the whole digestive system which results in damage to the gastrointestinal (GI) tract. IBD often emerges during adolescence and young adulthood. Maternal morbidity includes physical and psychological conditions that result from or are aggravated by pregnancy and have an adverse effect on a woman’s health, the baby’s health or both. Some women have health challenges that arise before or during pregnancy that could lead to complications. It is recommended for women to receive health care counseling before and during pregnancy. Compared to other developed countries, the United States has the highest rate of women dying of pregnancy related complications. During the past 25 years maternal mortality has been getting worse. African American women (AAW) with and/or without IBD are dying at significantly higher rates than other groups. This is linked to several factors, i.e., systemic, institutionalized, and structural racism in health-care delivery and subsequent toxic stress from people’s lived experiences of racism, limited knowledge about healthcare system function, lack of access to healthcare, (inclusiveness and insurance policies) all of which negatively impact these patients. African Americans (AAs) are also up to three times as likely to experience severe maternal morbidity: unexpected outcomes of labor and delivery, deficient or lacking prenatal care and social determinants of health like lack of transportation, adequate employment, limited literacy, and limited healthcare access contribute to poor health outcomes. Studies on IBD patients indicate Medicaid expansion is associated with reduced rates of maternal morbidity, particularly for African American Women (AAW) and increased access to preconception and prenatal services that make pregnancy and childbirth safer for parent and baby. Herein we examine the physiological changes of pregnancy in patients diagnosed with inflammatory bowel disease and their relationship perinatal outcomes and parenthood.

## 1. Introduction

Inflammatory Bowel Disease (IBD) encompasses two medically unsolved gastrointestinal disorders i.e., ulcerative colitis (UC) and Crohn’s disease (CD) and it presents with prolonged chronic relapsing and remitting inflammation of the digestive tract system.^[1–5]^ The aetiopathogenesis of IBD remains enigmatic but is believed to be due to multifactorial interplay between Western lifestyle, genetically susceptible individuals, the immune system, the intestinal change in microbiome symbiotic relationship (dysbiosis/dysbacteriosis), pollution, changing hygiene status, socioeconomic status and several other environmental exposure factors.^[6,7]^ The key differences between UC and CD is in tissue inflammation, damage, and prognosis, which suggest distinctive enteropathogenic processes responsible for their respective features, which at times may be challenging to interpret clinically and/or histologically.^[8].^ Typically, intestinal wall thickening is segmental in CD but continuous in UC.^[9,10]^ UC causes inflammation and ulceration of the mucosal and, to a lesser degree, the submucosal lining of the colon and rectum.^[9]^ Furthermore, CD differs from UC in that it may cause inflammation deeper within all layers of GI tract (transmural inflammation and skip lesions) and also may affect other non-GI organs through fistulation.^[11,12]^ Unfortunately, these features are obscure during the prodromal stages of the disease, confounding the treatment regimens.^[9,13].^

The incidence of IBD is alarmingly evolving in young adults worldwide.^[6,7]^ IBD incidence and prevalence is now contemplated to be an emergent global disease with health-care costs rising exponentially.^[6,14–16]^ The burden of IBD varies in different countries and locations, especially when compared between resource-limited,^[16–31]^ and rich countries.^[32,33]^ Estimated data suggest that 25% - 30% of cases with CD and 20% of patients with UC present early in life during adolescence and young adulthood.^[2,34–42]^ The magnitude of racial/ethnic and regional differences in the prevalence of IBD in the United States remains largely obscure warranting additional research.^[43,44]^ Until recently, IBD has predominantly affected whites, particularly Ashkenazi Jews but over the last three decades, IBD has “emerged” in minority communities.^[44–49]^

IBD patients may have questions about their fertility especially related to the effects of their medications and disease itself. Even more concerns are raised about the pregnancy course and their baby’s safety while having this chronic intestinal condition. The risks associated with IBD in pregnancy are significant numerous, including miscarriage, small-for-gestational-age infant, premature delivery, poor maternal weight gain, insomnia, and preeclampsia. Further, complications of labor and delivery include, placental abruption, and increased probability of cesarean delivery.^[50–58]^ During preconception counseling is paramount, potential for parents to get best maternal and infant health picture prediction which provides an opportunity to focus on the benefits of controlling disease activity. This preconception counseling is lacking for minority populations, especially AAW. A recent survey of adult gastrointestional (GI) program directors and trainees in the United States, reported that only one third of the trainees were satisfied with their level of IBD exposure, while more than half were uncomfortable dealing with IBD special situations including the management of pouch, stoma, pregnancy or postoperative patients.^[29,30]^ Involving a maternal-fetal medicine specialist early in the conversation provides more confidence for the patients as they make decisions about IBD management. The challenge of improving care to the AAW with IBD is best met with the power of online information, collaboration, and shared decision-making.^[31]^ Web-based resources were the first choice, more than any other, as an information aid for IBD clinical care and were selected by almost half of the trainees.^[29,30]^

Understanding the perinatal health care patterns of AAW with IBD patients will prepare us to manage the burden of IBD over time in this population. This review highlights a comprehensive, updated overview of the literature, the relative safety of medications used to treat IBD before, during and after pregnancy and breastfeeding, and summarizes the updated recommendations for immunosuppressant and biologics/ biosimilars to establish the current available perinatal IBD care in the AAW, contrast it with the increase in IBD and forecast the effects of IBD substantial costs for health care. The majority of patients are diagnosed early in life and the incidence continues to rise; therefore, the effect of IBD on health-care systems will rise exponentially. We also provide action tips to help safety guide the patients and clinicians during pre-, peri- and post pregnancy, and breastfeeding. Finally, we address identified neighborhood factors, education, healthcare access and quality, economic stability, and other personal and societal contexts that can help health care institutions work towards a common goal of achieving health equity, regardless of racial/ethnic background.

## 2. Core tip

Pregnant women in the health insurance coverage gap are eligible for Medicaid, but necessary preconception care is likely unavailable.^[6]^ Being uninsured prior to pregnancy is associated with a higher prevalence of risk factors that contribute to poor pregnancy outcomes, especially for AAW.^[58]^ AAW experience unacceptably high rates of poor maternal health outcomes, and a maternal mortality rate of three times that of their White counterparts.^[50–58]^ In fact, AAW with or without IBD are three times more likely to die from pregnancy or parturition than women of any other race in the United States.^[29–31]^ Both societal and healthcare related factors contribute to high rates of poor health outcomes and AAW maternal mortality. The profound risk that AAW face from conception, to birth, and into motherhood is structural racism.^[107–111^] Throughout the course of a pregnancy, AAW face a variety of healthcare related disadvantages compared to non-Hispanic or white women.^[75–78]^ The reasons for this may not be immediately obvious because AAW’s heightened risk of pregnancy-related death spans income and education levels. While AAW are at a higher risk for developing high blood pressure and fibroids during pregnancy, the predominant issue comes down to medical inequity and access to quality care. ^[59–67]^ AAW also face medical racism and clinical research that historically has excluded AAW.^[107,108]^ These risks and subsequent complications extend from pregnancy to childbirth and motherhood. In additional to IBD, these include postpartum cardiomyopathy, preeclampsia and eclampsia, causing AAW maternal mortality rates to be five times those of non-Hispanic women.^[66,78,134,135]^ AAW also are over two times more likely than non-Hispanic women to die of hemorrhage or embolism. The African American (AA) infant mortality rate in 2018 was 10.8 deaths per 1,000 live births, compared to 4.6 deaths per 1,000 live births for white babies. The infant mortality rate among AA families is still significantly higher than non-Hispanic families across income and education levels. Issues involving health equity and improving access to reliable and unbiased healthcare is of the utmost importance when it comes to decreasing the AA maternal morbidity and mortality rates.^[114].^

Moreover, uninsured AA is likely to delay prenatal care until they apply to enroll in Medicaid.^[58]^ Medicaid pays 40 percent of U.S. births and 65 percent of births to AA mothers as part of a comprehensive strategy to address the AA maternal health crisis. Medicaid under the Affordable Care Act have significant health coverage among AAW of reproductive age but does not cover preconception counseling and prenatal services that make pregnancy and parturition safer for parent and baby.^[112]^ Robust research evidence shows Medicaid expansion is associated with reduced rates of maternal death, particularly for AA.^[112]^ Closing the coverage gap would significantly improve Black maternal health.^[75]^ Overall, IBD affects people during their reproductive years and has emerged globally with rising prevalence and has become an emergent disease with healthcare costs rising at an exponential rate.^[6]^

The management of IBD in pregnancy, maternal and infant health is complex, demanding and challenging, especially to AAW.^[59–67]^ Poorly controlled IBD during pregnancy increases the risk of prematurity, low weight for gestation, and fetal loss.^[68]^ The default is to cease interventions during pregnancy and lactation, despite the known risk of fulminant disease, the commonest to pregnancy outcome.^[68–72]^

## 3. African American Women *versus* White American Women with Inflammatory Bowel Disease and Pregnancy

There are physiological changes during pregnancy including uterine expansion, increase in progesterone hormone level (that may delay gastric emptying) and anatomic compression and lower esophageal sphincter pressures, thereby increasing risk for reflux, nausea, early satiety, and constipation.^[73]^ A study evaluating infant outcome in women with IBD at an urban university, and tertiary-referral center, reviewed medical records with single births who received all of their gastroenterology and obstetrical care at the medical center over a 9-year period.^[74]^ In summary, this study reveal that IBD is not associated with a higher pre-term delivery, neonatal intensive care unit requirements or congenital abnormalities when compared to the general population. The study observed however that AAW had more infants with low birth weights than CAW (*P = 0.0459*). Coordinated gastroenterology and obstetrical care in women with IBD is critical to optimize infant clinical outcome.

**Figure 1.**
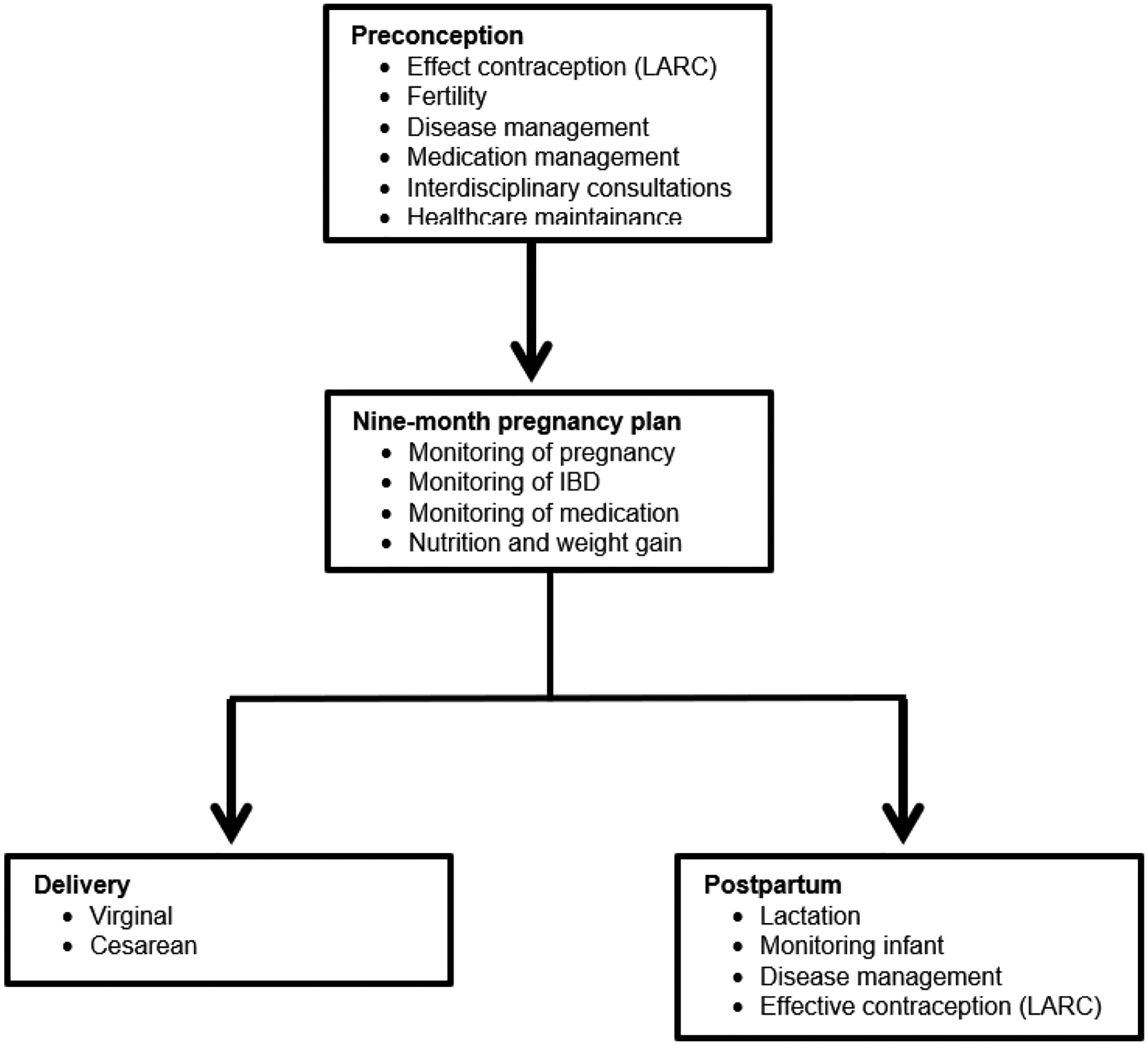
Overview of Inflammatory bowel disease in pregnancy clinical care pathway ^[32,89,90]^ **Abbreviations**: LARC, long-acting, reversible contraception; IBD, inflammatory bowel disease.

Considerable number of AA experience higher rates of maternal morbidity and mortality compared to US women of other racial/ ethnic groups. Lack of knowledge among both patients and physicians regarding reproductive health, especially in patients diagnosed with IBD is significant.^[75–78]^ The management of IBD during pregnancy is widely ascertained to all pregnant women but there is lack of knowledge among AAW regarding healthcare system inclusiveness and lack of health insurance negatively impacts these patients. They lack good control of disease and clinical remission at the time of conception which decreases the likelihood of quiescent disease during pregnancy necessary for having successful pregnancy outcomes. Closing the coverage gap would significantly improve black maternal health. Overall, IBD affects people during their reproductive years and has emerged globally with rising prevalence and has become an emergent disease with healthcare costs rising at an exponential rate.^[6]^ Remarkable progress has been made in the treatment of IBD in recent years resulting in successive launching of new pharmaceuticals (biologics / biosimilars), though understanding of safety use of each newly developed medication during pregnancy and their in utero effects have not been maintained. Studies have demonstrated increased concentrations of biological agents in infant’s blood stream multifold more than mother’s trough when administered in late gestation period. Relatively high concentrations of biological agents do not seem to cause adverse birth outcomes nor future developmental defects but may affect baby’s immune function. Therefore, special consideration should be given to vaccination schedule of the newborn babies whose mothers were treated with biologics / biosimilars in the late gestation period. A summarized overview of IBD in pregnancy clinical care pathways is depicted in Fig. 1.^[31,84,85]^ Importantly, good prenatal supplementing nutrition is advised to handle the extra body demands as the pregnancy progresses.^[79,80]^ The goal is to balance getting enough nutrients to support the growth of the fetus and maintaining a healthy weight.^[81]^ While all women need to increase intake of certain essential vitamin and nutrients (folic acid, Vitamin D & iron) to ensure a healthy pregnancy, the woman with IBD is at greater risk for these kind of deficits.^[81–83]^ This is especially true with fulminant IBD since bowel inflammation and diarrhea prevent proper absorption or the loss of nutrients. Fulminant IBD also reduce appetite and make food harder to digest. Prenatal dietary supplement guidelines are depicted in Fig. 3.

## 4. Conception - Fertility: Chances of becoming pregnant in African American Women patients with Inflammatory Bowel Disease

Women suffering from IBD have been shown to have similar fertility as the general population,^[84–92^] although some studies report that they have reduced fertility, especially when their disease is active, fulminant colitis.^[93,94]^ Clinically, fulminant colitis occurs in patients with severe UC who have more than 10 bowel movement per day, continuous mucosal bleeding, abdominal pain, distention, and acute, severe toxic symptoms including fever and anorexia.^[92,93,95]^ IBD patients can have normal fertility, however once UC patients have had a pouch surgery (restorative proctocolectomy with ileal pouch-anal anastomosis (RPC-IPAA)), they have an increased risk of infertility up to 3–4 fold.^[93–100]^ This increased infertility rate after RPC-IPAA is due to partial or total obstruction of fallopian tubes from the adhesions and scarring.^[45,99,100]^ Patients who have had laparoscopic RPC-IPAA have been shown to have less adhesions,^101^] and lower infertility rates.^[102,103]^ There are varying reports on the infertility rates among women with CD and UC. These differences are attributed to voluntary childlessness (VC), i.e., voluntary choice to not have children. In a meta-analysis of eleven studies, they found that in women with CD, fertility was decreased up to 44% *vs*. controls, however, further analyses divulged association to VC; they did not find any reduction in fertility in women with UC.^[49,104]^

The fact that knowledge on gastroenterological and obstetrical management of IBD has improved over the years, many patients still actively avoid pregnancy for fear of adverse maternal or neonatal outcomes. There is fear of adverse effects of pregnancy on the disease activity, of eventual IBD inheritance, or of an increased risk of congenital malformations. Indeed, though data indicate that fertility is hardly affected by the disease, there is conflicting knowledge about the impact of IBD on fertility, pregnancy and sexual function health and health care professionals (HCPs) do not sufficiently inform their patients about reproductive outcomes often associated with a higher risk of adverse pregnancy outcomes such as low birth weight, preterm birth, and spontaneous abortion. More research information on these topics is indisputably needed for IBD patients, especially AAW.^[105,106]^ Because of these uncertainty, many women with IBD may choose to remain childless due to a lack of IBD-specific reproductive knowledge.^[106]^ Another serious misconception challenges in planning pregnancy among AAW with or even without IBD is the maternal mortality crisis in the United States which emphasizes the validity behind this notion of systemic institutionalized structural racism.^[107,108]^ Racism is the relegation of people of color to inferior status and treatment based on unfounded myths, taboo and beliefs about innate inferiority, as well as unjust treatment and oppression, whether intended or not.^[109,110]^ Racism is not always conscious, explicit, or readily visible, often it is systemic and structural that is the systems’ platform/ scaffolding.^[111]^ Systemic and structural racism are forms of racism that are pervasively and deeply embedded in systems, laws, written or unwritten policies, and entrenched practices and beliefs that produce, condone, and perpetuate widespread unfair treatment and oppression of people of color, with adverse health consequences.^[112–115]^ Unfortunately causal pathways for health damages can take decades, even generations, may pass between exposure to systemic racism and evidence of its health damages, obscuring the connection experiences of racism contribute to racial or ethnic disparities in health by setting in motion various sequential causal pathways often difficult to detect their origins underlying unseen causes.^[113]^ There are several other examples of systemic institutional structural racism visible in policy discourse e.g. in political disempowerment, segregation, financial practices, environmental injustice, criminal justice system, historical examples, data aggregation etc. Strategies to dismantle systemic racism should be prioritized to addressing inequities in the key determinants of policy discourse such as health care.^116]^.

## 5. Sleep quality and pregnancy in African American Women patients with Inflammatory Bowel Disease

It has been long recognized that IBD patients suffer from poor sleep quality, only a handful of studies have evaluated the prevalence and risk factors associated with sleep disturbance and severity in IBD.^[118–122]^ Further, discrimination/racism is reported to be associated with poor sleep quality in pregnant AAW.^[123–125]^ Another study of patients who completed the Pittsburgh Sleep Quality Index (PSQI), the IBD questionnaire (IBDQ), the IBD-Disability Index (IBD-DI) questionnaire, and the Hospital Anxiety and Depression Scale (9-HADS) were analyzed using a multivariate regression model applied to assess independent risk factors of sleep disturbance among IBD-related variables, disability, quality of life, anxiety, and depression. The study investigated the sleep quality of 166 patients, finding 67.5% of them suffering from sleep disturbance. In particular, low quality of life, presence of disability and extraintestinal manifestations were identified as independent risk factors of sleep disturbance. The study discovered that all depressed patients were also affected by sleep disturbance, while found no difference in sleep disturbance between patients with or without anxiety state. However, a positive correlation was reported between both anxiety and depression scores and PSQI score (Spearman correlation: r = 0.31 and r = 0.38 respectively). The study showed that sleep quality is not directly associated with an active or inactive IBD state or with the ongoing treatment, but it is mostly correlated with the patients’ mood state, disability, and quality of life.^[126]^ Advisably, Gastroenterologists and Psychologists should join forces during clinical outpatients’ visits to evaluate emotional states for a better IBD management.

## 6. Pregnant African American Women Patients with Inflammatory Bowel Disease

**Figure 2.**
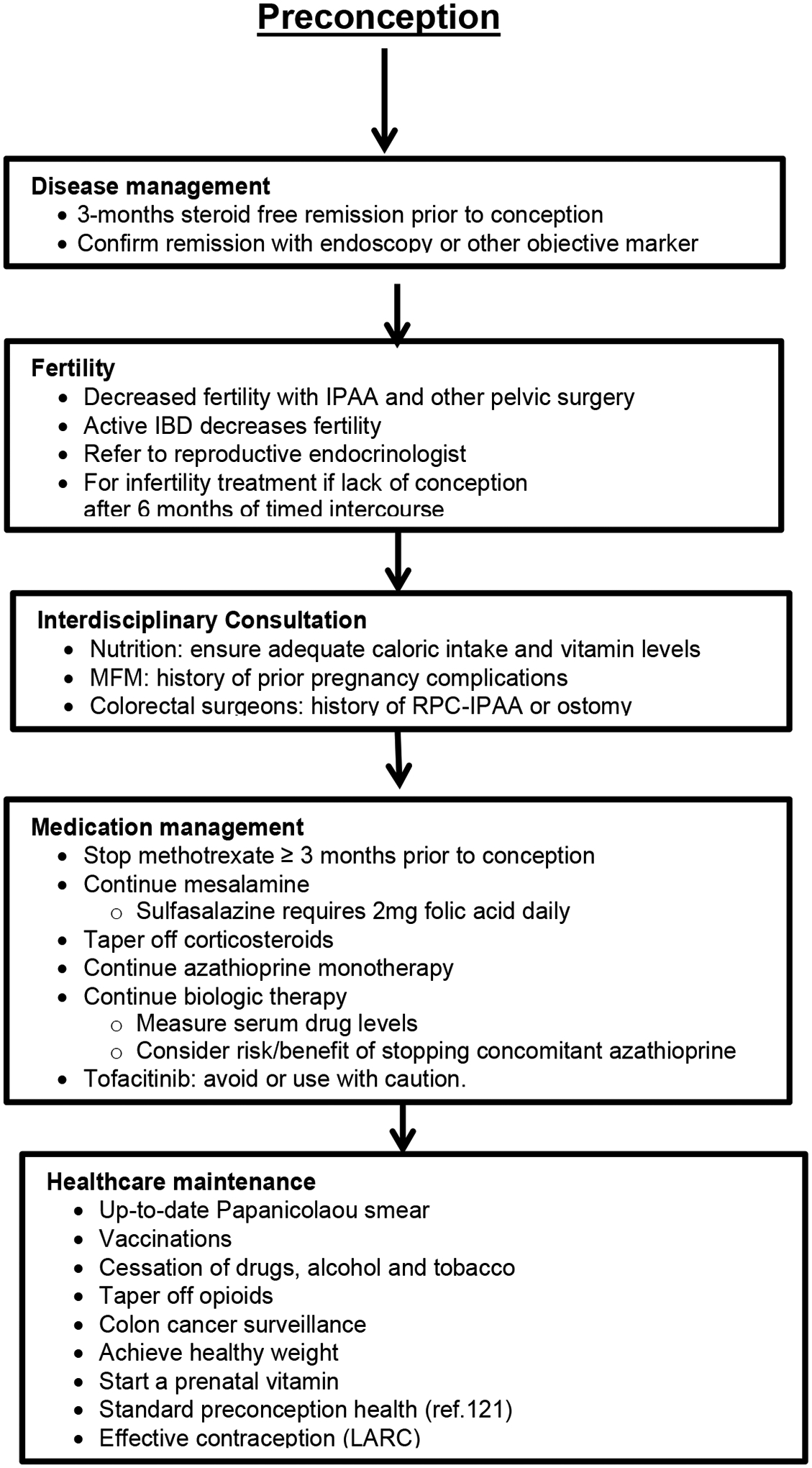
Pregnancy planning and conception ^[71,73,221]^ **Abbreviations**: ACOG, American College of Obstetricians and Gynecologists; LARC, long-acting, reversible contraception; IBD, inflammatory bowel disease; IPAA, ileal pouch-anal anastomosis; RPC-IPAA, restorative proctocolectomy with ileal pouch-anal anastomosis.

**Figure 3.**
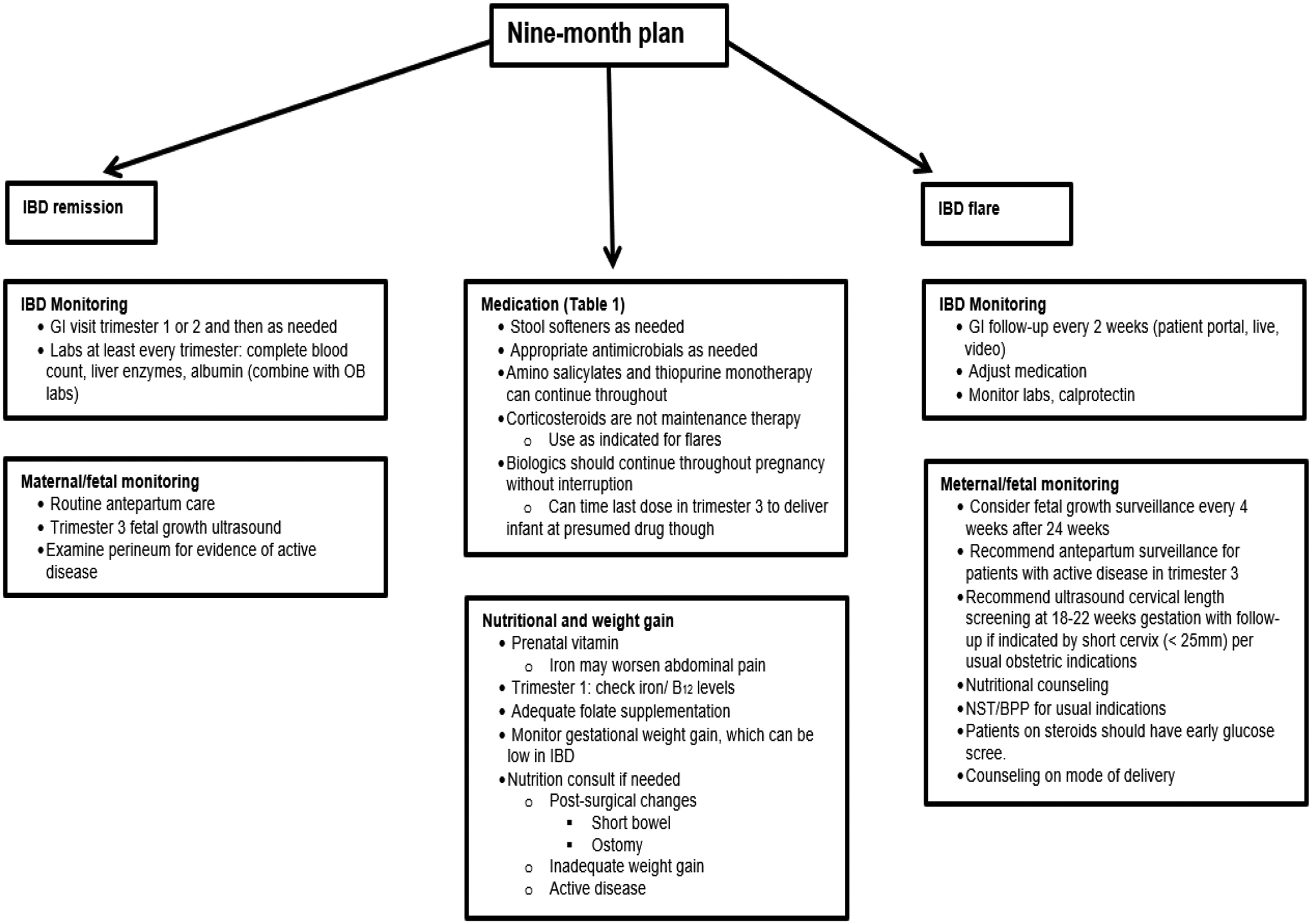
Nine-month plan^[73,186,198]^ **Abbreviations**: NST, Nonstress test; BPP, Biophysical profile; IBD, inflammatory bowel disease; NST/BPP; non-stress test/biophysical profile scan.

There are significant racial and ethnic differences in the incidence and temporal trends of IBD over the last few decades in the US population-based cohort.^[166]^ Studies suggest that migration and race influence the risk of developing IBD may be due to different inherent responses upon exposure to an environmental trigger in the adopted country.^[167]^ The prevalence of IBD is 96 cases per 100,000 person-years in African Americans.^[43]^ In the 1980s, a study from southern California, USA using the Kaiser Permanente Medical Care Program reported that African Americans had IBD prevalence approximately two-thirds of that of whites 29.8 cases per incidence of IBD among racial and ethnic groups in the US.^[166,168]^ In AAW with IBD face poor access to outpatient IBD specialist care which contributes to IBD-related emergency department (ED) visits.^[169]^ Strategies to increase specialist access may reduce the utilization of emergency services.^[170,171]^ IBD affects mainly young people who are often in the process of family planning.^[2,38,172]^ The patients with IBD commonly experience unexpected relapses of the disease and not a few IBD patients have disease flare during their pregnancy, childbirth, and nursing most likely due to endogenous cortisol levels which is more than double during pregnancy.^[173.174]^ Options for flare management and maintenance therapies during pregnancy planning and conception, pregnancy and lactation are depicted in Tables 1 & 2 ^[175–185]^ and Figure 1 and Figure 2.[31,72,84,85,193,194] Detailed nine-months plan is shown in Figure 3.[71,175,176,186,187] During pregnancy, many physiological changes occur in order to allow implantation and fetal growth. This is the reason why pregnancy represents a period of intense endocrine fluctuation and immune modulation.^[188]^ In previous years, it was thought that during pregnancy there was a rise in maternal immune tolerance; however, it is now emerging that immunological states fluctuate during these months on demand to meet various requirements.^[189]^ Insecurity of patients and their primary physicians concerning the disease course, successful pregnancy and the appropriate medication is to date still a challenge often followed by a decision against the pregnancy.^[190,191]^ A recent study comparing IBD and non-IBD pregnant women^[192]^ showed an improvement in the modulation of cytokine patterns during pregnancy in the first group. Indeed, IL-6, IL-8, IL-12, IL-17, and TNF-a proinflammatory cytokines significantly decreased after conception. During pregnancy itself, serum cytokine levels in patients with IBD subsequently remain relatively stable over the 40 weeks of gestation. On the other hand, Kim *et al*. and coworkers showed that a surrogate marker of bowel inflammation (i.e., Fecal Calprotectin (FC)), had higher levels in pregnant patients affected by IBD compared with controls, but it gradually decreased in the case-group.^[193]^ The opposite trend was observed in the control-group, demonstrating a slight gradual increase in their FC inflammation marker levels during gestation. As for babies born to mothers with IBD, the same study showed significantly higher FC levels compared with control babies from 2 to 36 months of age. The authors speculated that those babies may have been less able to achieve a balanced mucosal immunity or to establish an optimal intestinal barrier function. This fact is probably explained by a lower immune tolerance to commensal bacteria in babies born to IBD mothers, potentially leading to chronic mild intestinal inflammation due to a modification in the intestinal microbiota.

## 7. Pregnancy and Inflammatory Bowel Disease Care Coordination Team and Disparities

It is important to educate the young patients with IBD during family planning counseling.^[194–200]^ Low health literacy is present in IBD populations and more common among older AAs^[201–203]^ Opportunities exist for providing more health literacy-sensitive care in IBD to address disparities and to benefit those with low health literacy.^[203]^ Unfortunately, to date, racial disparities and racism severely impacts AAW during pregnancies in health and diseases while attending care coordination services.^[125,204,205]^ Fulminant colitis disease during conception and pregnancy in women with IBD increases the risk of pregnancy complications and adverse neonatal outcomes^.[185,206–208]^ Preferably, a pregnant patient with IBD should be monitored by both a gastroenterologist specialized in IBD and a maternal–fetal medicine (MFM) specialist, OB/GYN with assistance from nutritionists, lactation counselors, colorectal surgeons, and care coordinator as needed. Pregnancy planning and conception is depicted in Figure 2 [71,209,210] However, due to variations in access, availability, and preference, patients may receive their IBD care from a general gastroenterologist, nurse practitioner, physician’s assistant, surgeon, primary care provider, or even the emergency department. Similarly, obstetric care may be provided by a maternal fetal medicine (MFM) general obstetrician, midwife, and family practitioner. Painstakingly, many nulligravida and/ or nulliparous AAW have no one at all for much of the pregnancy.^[68,211]^ It is realizable that sizable number of patients and providers do not have access to IBD experts and MFM specialists, particularly outside of urban centers. However, any gastroenterologist, OB/GYN, or specialized physician’s assistant, nurse practitioner, or midwife can follow the Care Pathway to optimize outcomes in this population. Some patients are newly diagnosed with IBD during pregnancy and may be directed to a gastroenterologist after an emergency department visit, hospital admission, or visit with their primary care provider or obstetrician/ gynecologist (OB/GYN). Importantly, efforts to decrease institutional and interpersonal experiences of racial/ethnic discrimination and gendered structural racism would tremendously benefit the healthcare service and sleep quality of pregnant AAW,^[205,212–215]^ particularly during early pregnancy.^[124]^

## 8. The Inflammatory Bowel Disease Parenthood project and persistent barriers

**Figure 4.**
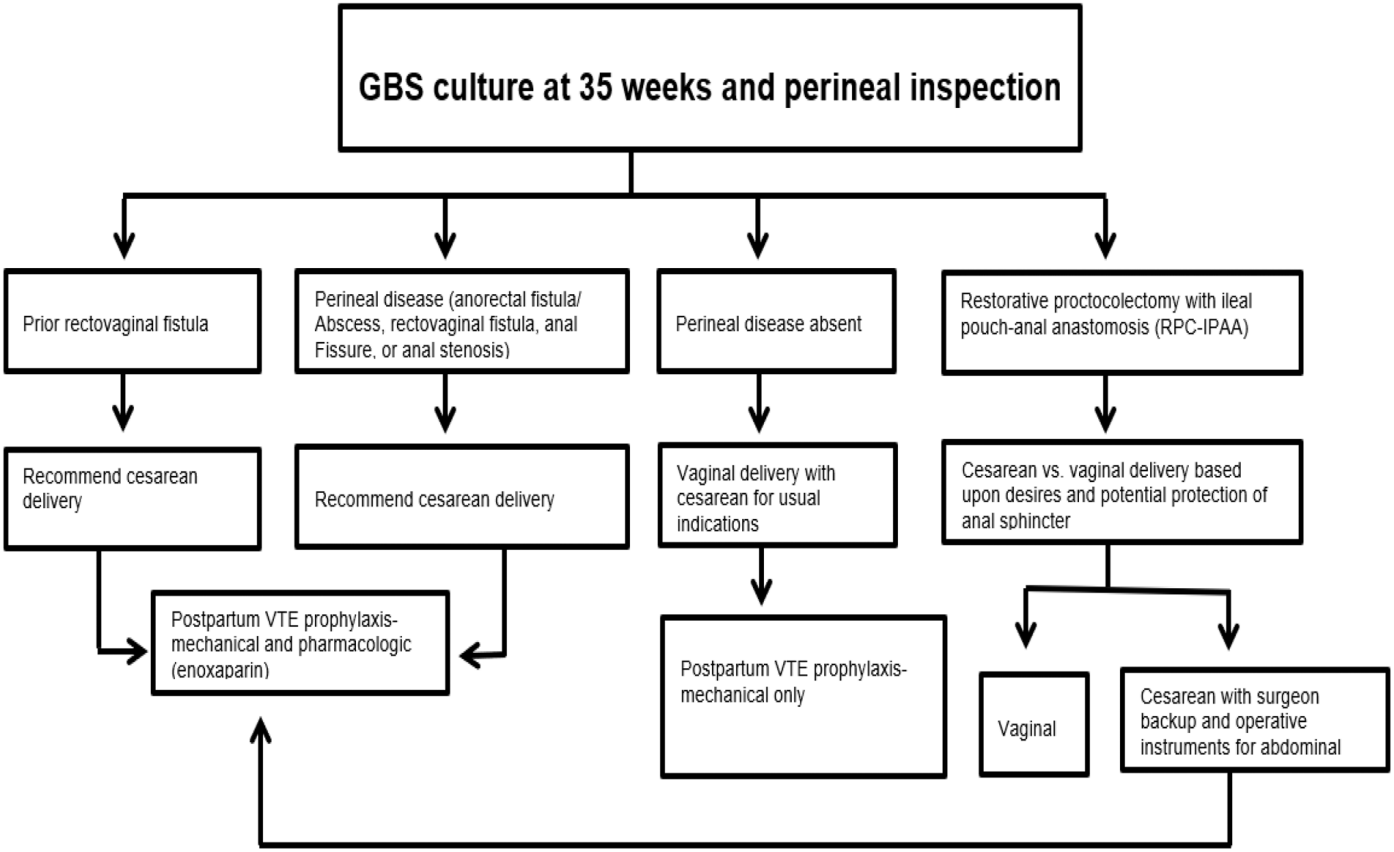
Decision algorithm for mode of delivery^[31,255–257]^ **Abbreviations:** GBS, group B streptococcus; VTE, venous thromboembolism; GBS, Group B streptococcus; VTE, Venous thromboembolic disease.

**Figure 5.**
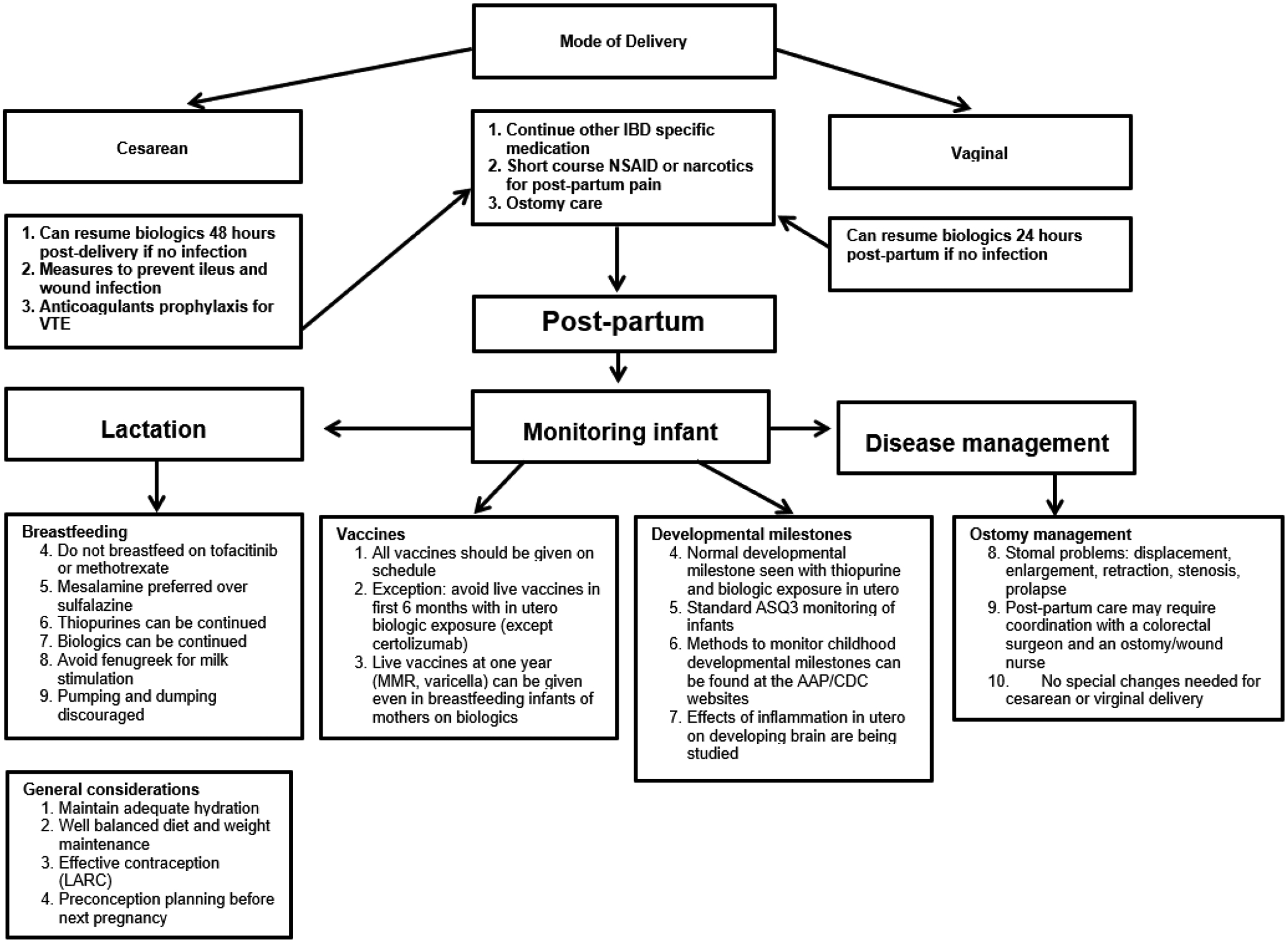
Post-delivery care for mother and baby^[258–260]^ **Abbreviations**: IBD, inflammatory bowel disease; LARC, long-acting, reversible contraception; MMR, measles, mumps, rubella; NSAID, nonsteroidal anti-inflammatory drug; VTE, venous thromboembolism; NSAID, nonsteroidal anti-inflammatory drugs; MMR, measles, mumps, rubella; AAP, American Academy of Pediatrics; CDC, Centers for Disease Control and Prevention.

Parenting mothers with IBD who are breastfeeding should follow standard nutritional recommendations.^[216–219]^ American Gastroenterological Association (AGA) runs an IBD parenthood project which provides information to women with IBD and how they can stay healthy and have healthy babies.^[71,186,220]^ The long-term safety of exposure to biologic/ biosimilar drugs in IBD patients during pregnancy has received attention. Recently, multicenter retrospective studies have been reporting on fertility health and pregnancy in these patients diagnosed with IBD.^[39,189,221–224]^ Further, there is shared information focused on disease characteristics, medication use, lifestyle, inadequate gestational weight gain, pregnancy outcomes and long-term health outcomes of children breastfed by women receiving biologic/ biosimilar therapies and effects of breastfeeding and development retrieved from mothers and medical charts.^[225–235]^ Assessment of adverse reactions to vaccinations, growth, infections, autoimmune diseases and malignancies are reported.^[236–242]^ Furthermore, the IBD Parenthood project further helps gastroenterologists provide care during all stages of family planning such as: groundbreaking medical research in IBD - a family affair, supporting IBD patients in family planning and pregnancy, how to care for IBD patients in pregnancy and, what is the IBD in Pregnancy Clinical Care Pathway? Overview of IBD in Pregnancy Clinical Care Pathway is herein depicted in Figure 1.^[31,84,85]^ Further, recommendation/ decision algorithm for mode of delivery – cesarean *vs*. vaginal is detailed illustrated in Figure 4.^[31,243–245]^ Post-delivery care for mother and baby is summarized in Figure 5.^[246–348]^

## 9. Pharmaceutics Strategies for Management of patients with Inflammatory Bowel Disease

To date, there is no definite curative pharmacological drug(s) exists for IBD, which may result in significant long-term comorbidity.^[249]^ The scientific evidence warrants assessing and providing expert opinion related to nutritional, psychological, and supportive care of women and their infants throughout the prenatal, antenatal, and infant periods.^[250,251]^ In recent years, the IBD community has witnessed the marketing of novel therapies involving orally administrated drugs that target key inflammatory signaling pathways.[71,186,187,225,252,253] However, these carefully designed drugs do not cure IBD but manages symptomatology temporarily; moreover, the active pharmaceutical ingredients have serious side effects, and a sub-group of patients are not responsive to them. Thus, given the personal and societal impact of this disease, this unmet need justifies the continued development of novel therapeutic strategies for the treatment of IBD.^[254,255]^ There are established clinical recommendation option guidelines for the flare management efficacy and safety for use of medical pharmaceuticals of IBD patients during pregnancy and lactation as summarized herein in Table 1 and Table 2.^[159–169]^.

## 10. Surgical therapy for Inflammatory Bowel Disease on Female Fertility

The standard curative surgical procedure for treating UC is pouch surgery, RPC-IPAA^.[256,–258]^ Women with refractory UC may require RPC-IPAA surgery which may increase risk of infertility.^[50,258–260^] Colonic Crohn’s disease (CD) called Crohn’s colitis (CC) is a relative contraindication to RPC-IPAA intervention because of higher rate of complications and pouch failure even in highly selected patient.^[82]^ Therefore, it is important to accurately categorize indeterminate colitis (IC) into authentic either UC or CD prior to surgery.^[261–264]^ We identified 16 observational studies of which ten studies were included in meta-analyses, of which nine compared women with and without a previous IBD-related surgery and the other compared women with open and laparoscopic RPC-IPAA. Of the ten studies included in meta-analyses, four evaluated infertility, one evaluated assisted reproductive technology (ART), and seven reported on pregnancy-related outcomes. Seven studies in which women were compared before and after colectomy and/or RPC-IPAA were summarized qualitatively, of which five included a comparison of infertility, three included the use of ART, and three included other pregnancy-related outcomes. One study included a comparison of women with and without RPC-IPAA, as well as before and after RPC-IPAA, and was therefore included in both the meta-analysis and the qualitative summary. All studies were at high risk of bias for at least two of Chochran risk-of-bias tool domains. The Chochran risk of bias tool covers six domains of bias i.e., selection bias, performance bias, detection bias, attrition bias, reporting bias, and other bias.^[265–269]^ According to the latest AGA, American Society of colon and Rectal Surgeons (ASCRS) and European Crohn’s and Colitis Organization (ECCO) guidelines on reproduction, UC without previous pelvic surgery and inactive CD do not impair fertility.^[178,270]^ Conversely, active CD may impair fertility via multiple factors such as pelvic inflammatory disease (PID) which includes fallopian tube inflammation (salpingitis) and/or ovaries (oophoritis) or Inflammation of the fallopian tubes and ovaries simultaneously (salpingoophoritis).^[271]^ Different considerations should be made in UC patients who underwent RPC-IPAA, which seems to increase the risk of infertility by approximately threefold mainly due to tubal dysfunction caused by adhesions.^[97,99]^ Women with RPC-IPAA mostly suffer a reduction in the probability of conception rather than complete infertility.^[272,273]^ Because complications during pregnancy and delivery are rare, caesarean section should be based mainly on obstetric indications.^[274,275]^ Considering all the above possible factors lead to infertility, patients with IBD may be referred to ART earlier than the general population, even after only six months of attempts.^[271]^ It is still not clear to date if the ART success rate in IBD patients differs from the general population.

## 11. Concluding remarks

In spite of the fact that knowledge on gastroenterological and obstetrical management of IBD has greatly improved over the years, many patients still actively avoid pregnancy for fear of adverse maternal or neonatal outcomes, of adverse effects of pregnancy on the disease activity, of eventual IBD inheritance, or of an increased risk of congenital malformations. Although data hints that fertility is hardly affected by the disease, a reduced birth rate is nevertheless observed in patients with IBD. Moreover, physicians often showed concerns about starting IBD medications before and during pregnancy and did not feel adequately trained on the safety of IBD therapies. IBD-expert gastroenterologists and gynecologists should discuss pregnancy and breastfeeding issues with patients in order to provide appropriate information; therefore, pre-conception counseling on an individualized basis should be mandatory for all patients of reproductive age to reassure them that maintaining disease remission and balancing the eventual obstetrical risks is possible. A healthcare system that is not inclusive is problematic in certain countries including the United States and a substantial number of women are not counseled in a timely manner and those with IBD are as well ineffectively counseled of immunosuppressive therapy on fertility health and pregnancy.^[276]^ This largely impacts AAW linked to structural racism in health care delivery and subsequent toxic stress of racism, knowledge about healthcare system function, inclusiveness and insurance policies negatively impacts these patients. IBD control prior to conception and throughout pregnancy is the cornerstone to successful pregnancy management in these patients. Although the woman with IBD possesses a greater potential for a complicated pregnancy, the majority of these patients will experience an uneventful normal pregnancy. It is important to educate the young patient with or without IBD during family planning counseling. Conception at a time when IBD is quiescent offers the greatest likelihood of an uncomplicated pregnancy. Physicians must recognize and inform their patients that most medications that are necessary to suppress the disease should be continued throughout pregnancy, Table 2. Although generalities can be made regarding the management of pregnant women with IBD, the individual patient may need specifically tailored therapy for her individual case. There is no evidence to suggest that babies born to mothers with IBD regardless of medication exposure have any developmental delays. Recommendations on monitoring childhood developmental milestones can be found at the American Academy of Pediatrics and Centers for Disease Control and Prevention websites.^[228,277]^ The PIANO data on developmental milestones support the lack of negative effect of IBD medications on development.

## Figures and Tables

**Table 1.** Options for flare management^[(187,196,200]^

Medical Treatment	Safety and Recommendations in Pregnancy	Safety and Recommendations in Breastfeeding
**Aminosalicylates** (mesalazine, sulfasalazine, balsalazide, olsalazide)	No increased obstetrical risk. Always recommended (formulation without dibutylphthalate are preferable and, if sulfasalazine is used, suggestion to supplement with folate)	Safe and must be discontinued only in case of neonatal severe bloody diarrhea.
**Corticosteroids**	Concerns about teratogenic effects, such as cleft lip or palate. Recommended only in case of active flares	Recommended to breastfeed babies 4 h after taking corticosteroids
**Antibiotics** (metronidazole and ciprofloxacin)	Concerns about teratogenic effects, such as cleft lip or plate.Recommended only after the first trimester of gestation.	Recommended to breastfeed babies 12–24 h after metronidazole and 48 h after ciprofloxacin intake. A short-term antibiotic regimen must be preferred
**Thiopurines** (azathioprine or 6-mercaptopurine)	Slight increase in preterm deliveries. Recommended as monotherapy	Advisable, no a higher risk of physical or developmental anomalies in newborns
**Methotrexate**	Strong teratogenicity and abortive effects. Never recommended in pregnancy	Contraindicated
**Cyclosporine**	No data on pregnant women available, only recommended as rescue therapy for acute severe steroid-refractory ulcerative colitis	Contraindicated
**Antibiotics** (metronidazole and ciprofloxacin)	Concerns about teratogenic effects, such as cleft lip or plate.Recommended only after the first trimester of gestation.	Recommended to breastfeed babies 12–24 h after metronidazole and 48 h after ciprofloxacin intake A short-term antibiotic regimen must be preferred
**Thiopurines** (azathioprine or 6-mercaptopurine)	Slight increase in preterm deliveries. Recommended as monotherapy.	Advisable, no a higher risk of physical or developmental anomalies in newborns.
**Anti-TNFα agents** (infliximab, adalimumab, golimumab and certolizumab)	Evidence of crossing the placenta, except of certolizumab Recommended stopping around the 24th week of gestation, if the case permits.	Safe due to their transmission in breast milk only in small amounts and deactivation by neonatal digestion enzymes
**Vedolizumab** and ustekinumab	Should be avoided due to their transmission across the placenta and partial lack of data in pregnancy. Can eventually be prescribed only as an ultimate alternative	Safety data are still missing, so their use is not recommended
**Tofacitinib**, **filgotinib and upadacitinib**	Contraindicated due to the complete lack of data in pregnancy.	Safety data are still missing, so their use is not recommended.

**Abbreviations**: CRP, C-reactive protein; CT, computed tomography; ESR, erythrocyte sedimentation rate; MRI, magnetic resonance imaging, IBD, inflammatory bowel disease.

**Table 2.** Inflammatory bowel disease maintenance therapies during pregnancy and lactation^[187,196,200]^

Laboratory Values	Endoscopy	Radiology imaging	Surgery	Medication
Standard IBD laboratory values chacked	Perform for strong indications:	MRI and CT have similar diagnostics accuracy for assessing IBD	Surgery intervention may be needed:	Mange similar to nonpregnant IBD Patients
Trends for CRP and ESR may be helpful Posibly elevared	-Determining IBD disease activity	Gadolinium should be avoided in pregnancy	- Acute refractory colitis - Perforation	Exceptions: -Thiopurine-naïve patients avoid first start in pregnancy due to concerns for distinctive rare adverse reactions
Fecal calprotectin	-When results will change management	The cumulative radiation exposure of a single CT scan (about 50 mGy) is below the level of concern	-Abscess -Severe hemorrhage	Methotrexate contraindicated
Serum drug concentration	Flexible sigmoidoscopy is preferred oner pancolonoscopy when possible; can be performed unsedated, and in any trimester	Ultrasound, where available is appropriate for terminal ileal disease	-Bowel obstruction	Tofacitinib: avoid due to limited human data
Posibly elevared ESR CRP				
Alkaline phosphatase (elevated in lactation)				
Reduced in pregnancy Hemoglobin Albumin				

**Abbreviation:** Anti-TNFα, tumor-necrosis factor-α

## Practice Point Areas

- Profound hormonal changes may impact onset of prodromal IBD and/or worsen active IBD in pregnant women particularly in AAW.
- Pregnant women with IBD are more likely than the general pregnant population to experience adverse maternofetal outcomes, especially at the time of conception and during pregnancy.
- Pregnant women with IBD are more likely than the general pregnant population to experience adverse maternofetal outcomes, especially at the time of conception and during pregnancy.
- Salivary cortisol and alpha-amylaseare novel biomarkers of stress tied to triggering of latent IBD, reflecting the hypothalamic-pituitary-adrenal axis and sympathetic nervous system, respectively.^[278]^
- Our literature reviews show that sleep quality is not directly associated with an active or inactive IBD state or with the ongoing treatment, but rather it is mostly correlate with the patients’ mood state, disability, and quality of life.^[118,126,279]^
- Gastroenterologists, obstetricians, and psychologists should join forces during clinical outpatients’ visits to evaluate emotional states for a better IBD management.

## Research Agenda/Plan

- Consequences of racism before, during and after pregnancy complicating IBD.
- Action is needed to have unfettered access to healthcare and facilitate early detection of IBD, especially in AA.
- The effect of chronic disease need to be evaluated in future studies by including a comparative group of pregnant women with active IBD.^[278]^
